# Double-Blind Clinical Trial of Arginine Supplementation in the Treatment of Adult Patients with Sickle Cell Anaemia

**DOI:** 10.1155/2019/4397150

**Published:** 2019-02-03

**Authors:** Renata M. N. Eleutério, Francisco O. Nascimento, Tamara G. Araújo, Marilena F. Castro, Tarcísio P. Almeida Filho, Pedro A. Maia Filho, José Eleutério, Darcielle B. D. Elias, Romélia P. G. Lemes

**Affiliations:** ^1^Graduate Programme in Development and Technological Innovation of Medicines, Federal University of Ceará, Fortaleza, Brazil; ^2^State Blood Centre of Ceará (HEMOCE), Fortaleza, Brazil; ^3^Graduate Programme in Pharmaceutical Sciences, Federal University of Ceará, Fortaleza, Brazil; ^4^Motherhood and Child Department, Federal University of Ceará, Fortaleza, Brazil

## Abstract

**Background:**

Sickle cell anaemia (SCA) is the most prevalent monogenic disease in Brazil. In SCA, haemoglobin S (HbS) is formed, which modifies red blood cell morphology. Intravascular haemolysis occurs, in which free Hb and free radicals degrade nitric oxide (NO) and release arginase, which reduces arginine levels. Because arginine is a substrate for NO formation, this decrease leads to reduced NO (vasodilator) synthesis. SCA treatment uses hydroxyurea (HU) to maintain high foetal haemoglobin (HbF) levels and reduces HbS to avoid haemolytic episodes.

**Objective:**

To analyse the efficacy of L-arginine as an adjuvant in the treatment of SCA patients.

**Setting:**

The State Blood Centre of Ceará, Brazil.

**Methods:**

This was a randomized double-blind clinical study of adults with SCA with continuous use of HU at the State Blood Centre of Ceará. The clinical study enrolled 25 patients receiving HU + L-arginine (500 mg) and 25 patients receiving HU + placebo. The treatment was carried out over four months. Laboratory tests were performed to determine the levels of the following: (1) complete blood count; (2) nitrite + nitrate; (3) HbF; and (4) reticulocytes. The clinical experiments were performed by a haematologist. The main outcome measures were nitrite and pain.

**Results:**

Statistical analysis showed that the levels of NO were increased in the study group, and there was also a reduction in pain frequency using a pain frequency scale by day, week, and month. The levels of nitrite plus nitrate in the group receiving placebo plus HU did not change among the times evaluated (38.27 ± 17.27 mg/L, 39.49 ± 12.84 mg/L, 34.45 ± 11.25 mg/L,* p* >0.05), but in the patients who received supplementation with L-arginine plus HU, a significant increase in nitrite plus nitrate levels was observed between M0 and M4 (36.55 ± 20.23 mg/L versus 48.64 ± 20.63 mg/L,* p* =0.001) and M2 and M4 (35.71 ± 15.11 mg/L versus 48.64 ± 20.63 mg/L,* p* <0.001). It is important to note that the increase in nitrite plus nitrate levels occurred only in the fourth month of follow-up of patients in the treatment group, showing that at least 4 months of supplementation with L-arginine is necessary to show an increase in these metabolites in the serum.

**Conclusion:**

The use of L-arginine as a coadjuvant in the treatment of sickle cell anaemia may function as a potential tool for pain relief, consequently improving the life of patients.

## 1. Introduction

Sickle cell anaemia (SCA) is a genetic disease caused by a point mutation in the beta globin gene in which a glutamic acid is replaced by a valine, resulting in the synthesis of a new haemoglobin, haemoglobin S (HbS), which forms polymers in the red blood cells at low oxygen tension. It also makes membranes more rigid, thus modifying membrane morphology and function [1]. In this way, the transport of oxygen is compromised, and a deposition of these red cells in the endothelial wall occurs, which can lead to chronic inflammation, difficult microcirculation, pain, hospitalization, and stroke [2, 3].

Arginine is an essential compound for the formation of nitric oxide (NO), which is a potent vasodilator. It is found in reduced levels in SCA patients, which may cause impairment in the microcirculation, recurrent pain, and hospitalization. Studies have shown that, with increased L-arginine availability, there is greater NO synthesis, which leads to improved microcirculation and patient clinical profiles [2–5].

This is because L-arginine is a substrate for NO production. During haemolysis, arginase, an enzyme that metabolizes L-arginine and contributes to a decrease in NO concentration in SCA patients, is released. Other studies have demonstrated that NO is a cofactor for the enzyme guanylate cyclase, which is responsible for the conversion of guanosine triphosphate (GTP) to cyclic guanine monophosphate (cGMP). cGMP is responsible for smooth and vascular muscle relaxation and vasodilation [6–8].

Currently, to reduce haemolytic episodes, pain, and vessel occlusion, SCA treatment uses hydroxyurea (HU), which is used to maintain high foetal haemoglobin (HbF) levels and consequently reduce haemoglobin S (HbS) concentrations. Other positive effects associated with the use of hydroxyurea may be attributed to nitric oxide. There are several mechanisms involved in the production of nitric oxide from HU, such as the catalase-mediated pathway, urease-dependent formation, and NO production catalysed by horseradish peroxidase. More recently, the role of HU in inducing eNOS (NO endothelial synthetase) for NO production in endothelial cells has also been demonstrated [9–12]. In fact, this drug has been shown to be useful in relieving pain in patients with sickle cell anaemia, and the vasodilatation resulting from the formation of hydroxyurea-derived NO may be one more mechanism by which HU can benefit these patients [13]. Therefore, investigating whether L-arginine supplementation can enhance the effects of HU becomes mandatory since this amino acid plays an important role as a substrate for NO production.

In this context, the aim of this study was to analyse whether there is an increase in nitric oxide levels, measured by nitrite plus nitrate levels, in patients who use HU plus L-arginine as supplemental treatment compared to the nitric oxide levels in patients using HU plus placebo.

## 2. Materials and Methods

The clinical trial was a randomized, double-blind, and placebo-controlled study. The patients were randomized by the GraphPad prism program. Fifty adult patients of both sexes with a clinical and molecular diagnosis of SCA in their follow-up periods at the Blood Centre of the State of Ceará (HEMOCE) participated in the study. The inclusion criteria included adult patients using HU and baseline data according to the criteria of Ballas (2012): absence of painful episodes and/or intercurrent illnesses, such as infections and inflammations in the four weeks prior to the study; absence of hospital admissions in the last 2-3 days prior to study; and the absence of blood transfusion in the four months preceding the study [14]. The exclusion criteria were absence of a molecular diagnosis of SCA, pregnancy, presence of renal disease, not using HU, and a history of transfusion in the last six months.

Patients who participated in the study are seen at the haemoglobinopathies clinic regularly to continue their treatment with hydroxyurea. We use the moment when they returned to carry out the instruction and the trial.

The groups were separated in a randomized, double-blind trial in which only the main author had the information regarding the groups and the respective patients. Therefore, there are no other names in contributorship statement/acknowledgements. The physician who cared for the patients is a coauthor of this article.

Patients were stratified into two groups: (1) the first group consisted of 25 subjects who used HU + L-arginine at a dose of 500 mg/day and (2) the second group consisted of 25 subjects taking HU + placebo (starch). The HU doses ranged from 500 to 1500 mg/day and were not modified during the trial. Biological samples were obtained at specific timepoints: (1) before receiving supplementation with placebo or L-arginine (M0); (2) two months after placebo or supplementation with L-arginine (M2); and (3) four months after placebo or supplementation with L-arginine (M4). The arginine dose was defined based on an earlier study performed by the same group [5]. The trial time was determined by the clinical staff of the Blood Centre of the State of Ceará. It was not possible to use a group that used only arginine because of the treatment protocol of the institution.

The outcome measures were the nitric oxide dose measured by nitrite plus nitrate levels using a nitrite/nitrate, colorimetric assay kit, Roche®; foetal haemoglobin levels measured by HPLC; and reticulocyte and complete blood count. To measure pain frequency, a time scale was used to stratify the patients. The scale divided the patients into 5 pain categories: never (absence of pain), every day, every week, every month, and every year. As was done for pain assessment, a scale was created to measure the frequency of hospitalizations. The patients were stratified into the following: never hospitalized, hospitalized 5 years ago, hospitalized 3 years ago, and hospitalized this year. The other clinical and historical data of the patients were obtained from medical records.

Statistical analyses were performed using IBM SPSS 21 Statistics. For the analysis of the normality of the data, the Shapiro-Wilk test was used. For the analysis of the quantitative variables, a Mann-Whitney test or two-way repeated measures ANOVA were used. For the analysis of categorical variables, the Pearson's chi-square test or Fisher's test was used. Values of* p* <0.05 were considered significant.

The study was approved by the Ethics and Research Committee of the Federal University of Ceará with number 1.292.517, and it was registered in the Brazilian Registry of Clinical Trials with the number U1111-1201-6937. The patients were required to sign an informed consent form.

## 3. Results and Discussion

### 3.1. Patients

The mean age of patients enrolled in the study was 28 years, and a similar mean was observed in both of the groups, the placebo group and the treatment group. Most of the patients were female (52%), and the female-male ratio was approximately 1.1:1. Gender and age were matched between patients in the placebo and study groups. In addition, there was no difference in clinical manifestations or time of treatment with HU between the groups studied, showing that the baseline characteristics were similar between the groups (Table 1). The duration of HU use before the trial ranged from 2 to 180 months with a median of 48 months. No adverse effects were observed during the 4 months of the trial. There was no significant difference in the nitrite/nitrate levels in the serum based on the HU doses (*p* = 0.643).

### 3.2. Haematological Parameters

In patients for whom haematological data were available, no significant difference was observed between patients in the placebo group and in the study group, showing that supplementation with L-arginine had no influence on the haematological parameters evaluated when compared to patients receiving placebo (Table 2).

### 3.3. Measurement of Nitrite Plus Nitrate and Analysis of the Frequency Pain

**Figure 1:**
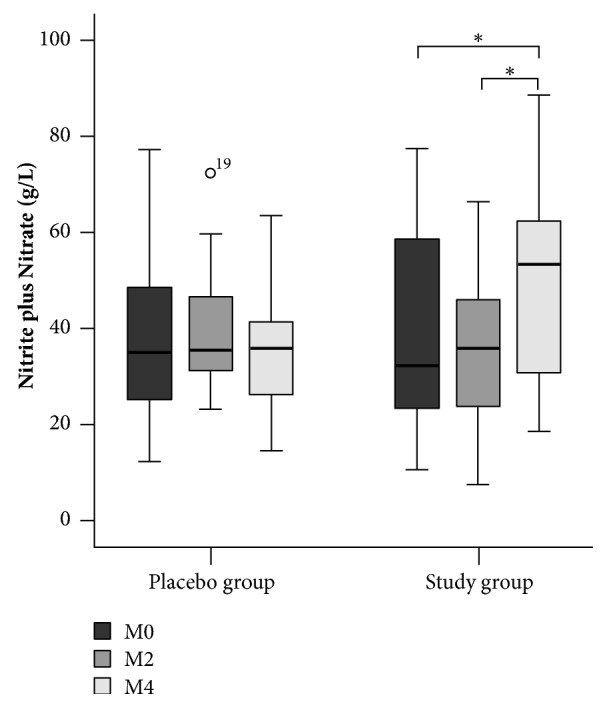
Comparison between the levels of nitrite plus nitrate in the different treatment groups and at the different moments evaluated. Two-way repeated measures ANOVA. *∗p* ≤0.001 in the comparison between M0 and M4 and M2 and M4. M0 (timepoint before receiving placebo or L-arginine supplement); M2 (timepoint two months after receiving placebo or L-arginine supplement); M4 (timepoint four months after receiving placebo or L-arginine supplement).

The levels of nitrite plus nitrate in the group receiving placebo plus HU did not change among the times the levels were evaluated (38.27 ± 17.27 mg/L, 39.49 ± 12.84 mg/L, 34.45 ± 11.25 mg/L,* p* >0.05), showing that placebo had no influence on nitrite plus nitrate levels. Patients who received supplementation with L-arginine plus HU showed a significant increase in nitrite plus nitrate levels between M0 and M4 (36.55 ± 20.23 mg/L versus 48.64 ± 20.63 mg/L,* p* =0.001) and M2 and M4 (35.71 ± 15.11 mg/L versus 48.64 ± 20.63 mg/L,* p* <0.001) (Figure 1). It is important to note that the increase in the nitrite plus nitrate levels occurred only in the fourth month of follow-up of the patients in the treatment group, indicating that at least 4 months of supplementation with L-arginine is necessary to show an increase in these metabolites in the serum.

The chi-square test showed a reduction in pain frequency in the study group compared to that of the placebo group (*p* =0.027).  Table 3 shows a reduction in the frequency of pain in patients in M4, showing that patients who presented pain every day started to have pain every week or every month. Interestingly, the change in pain frequency coincided with the increase in nitrite plus nitrate levels, suggesting a possible relationship between these two variables.

To the best of our knowledge, this is the first placebo-controlled double-blinded, randomized clinical trial that has tested the therapeutic potential of oral L-arginine supplementation over a four-month period in adult patients with SCA who are undergoing a classic treatment with basal HU. Our results suggest that this association may be beneficial for patients with sickle cell anaemia since a reduction in the frequency of pain was observed in patients who used HU plus L-arginine supplementation. This reduction was dependent on treatment time as observed from serum levels of nitrite plus nitrate, indicating a strong relationship between pain frequency and serum levels of these metabolites.

Nitrite and nitrate are stable products of NO metabolism that are accessible for quantification. They are present in both blood samples and urine samples. The measurement of nitrite and nitrate is considered the most adequate and practical way to evaluate the synthesis of NO in vivo. The colorimetric method used in our study is based on the Griess reaction, which involves the indirect determination of NO through the spectrophotometric measurement of its stable decomposition products, NO^2-^ and NO^3-^ [15].

It was not possible to include a group not being treated with HU because the hospital in which the treatment was performed treats patients with SCA with HU. However, in an earlier study in the same city, but in another hospital, it was possible to determine that the mean nitrite/nitrate level of patients was 25.63 mg/L, which was well below the indices in our study [16].

Previous studies conducted by our research group have shown an increase in nitrite levels in patients using HU in combination with L-arginine; however, we did not evaluate the influence of L-arginine on the frequency of pain in these patients [5]. Lopez et al. (1996) showed that levels of nitric oxide metabolites were inversely correlated with pain scores in patients with SCA with vaso-occlusive crises, suggesting the role of NO in pain [17]. Another study using inhaled NO also showed a decrease in pain score in patients with SCA who inhaled NO, reinforcing their role in pain relief [18]. In 2013, in a randomized, placebo-controlled trial using oral or intravenous L-arginine hydrochloride as a source of NO, Morris et al. (2013) demonstrated a 54% reduction in opioid use in children with SCA. In this same trial, the reduction in pain scores in children was observed in the study group versus the placebo group [19]. Cox et al. (2018) showed that arginine and citrulline treatment in children did show a benefit in measures of endothelial function compared with the same measures at baseline [20].

Our results are consistent with previously published data indicating that the use of L-arginine may reduce the frequency of pain in patients with sickle cell anaemia, demonstrating that it is a potential tool for improving the quality of life of these patients. This may be partly due to the increased serum levels of nitrite plus nitrate, which reflects increased NO, a known lacking vasodilator in patients with SCA that is associated with pain [16, 21].

Despite the few studies using L-arginine as a source of NO in SCA, many studies have shown the benefits of NO. For example, it has been reported that NO can be produced by the vascular endothelium from L-arginine and is able to regulate normal vascular tone and inhibit platelet activation, haemostatic activation, and the expression of adhesion molecules such as VCAM-1, which is a marker of endothelial activation that is implicated in binding to *α*4*β*1 adhesion molecules present in sickle red blood cells [22, 23]. The adhesion of sickle red blood cells to the endothelium, in turn, has been implicated in the initiation and propagation of vaso-occlusive events, as demonstrated in previous studies using an in vitro adhesion assay or an ex vivo perfusion model in rats [24]. Additionally, NO has been shown to reduce the adhesive properties of neutrophils to the vascular endothelium in SCA, an important event for the development of vaso-occlusive crises [25, 26].

In addition, Villagra et al. (2007) also reported that sildenafil, a phosphodiesterase 5 inhibitor known to potentialize NO-mediated signalling, was able to reduce platelet activation, which supports a role for NO-based therapeutics since platelet activation may play an important role in the adhesion of sickle red blood cells to the vascular endothelium [27]. Interestingly, platelet activation has been found to be elevated in patients with steady-state SCA, and this elevation is even greater during vaso-occlusive crises [26]. Taken together, these reports suggest that source-independent NO can benefit patients with sickle cell disease by acting on several cellular components involved in the genesis of vaso-occlusive crises. Here, we used L-arginine as a source of NO; L-arginine is a safe and effective amino acid that is reported to have narcotic-sparing effects that may be a beneficial complement to the treatment of patients with SCA [19].

Relatively recent studies have shown that the decomposition products of NO, nitrite and nitrate, can be recycled in vivo for the production of NO, representing an alternative source of this gas. This process occurs mainly under conditions of hypoxia in which the activity of the oxygen-dependent NOS enzyme is compromised [28]. In this context, we hypothesized that supplementation with L-arginine may, in addition to serving as a substrate for NO production by the NOS-dependent pathway, increase the supply of nitrite and nitrate to function as a storage pool that is available for NO production in conditions of low oxygen tension, such as the conditions that occur during vaso-occlusive crises. The increased NO levels may not only prevent vaso-occlusive crises but also attenuate or even resolve them. It is important to note that, during our study, no patient receiving HU plus L-arginine supplementation experienced complications of the disease.

## 4. Conclusions

In summary, our data indicate a strong relationship between increased levels of NO metabolites and decreased pain frequency in patients with SCA, which supports the use of L-arginine supplementation as an adjuvant in the treatment of these patients. These data are the first to demonstrate this relationship in adult patients with SCA; however, more studies are needed with a larger number of individuals and longer follow-up to evaluate the safety and efficacy of long-term supplementation with L-arginine.

## Figures and Tables

**Table 1:** Baseline characteristics of the patients.

	** Placebo Group**		** Study Group**		***p*-value**
**Gender (n/**%**)**					** >0.99**9^**α**^
Female	13	52,0%	13	52,0%	
Male	12	48,0%	12	48,0%	
**Age (Years)**	28		28		** 0.84**3^**γ**^
**HU doses (mg/day)**	1040		1070		
**Time of use of HU (months)**					
02 – 48	17	68,0%	14	56,0%	** 0.67**8^**α**^
49 – 96	6	24,0%	8	32,0%	
>97	2	8,0%	3	12,0%	
**Hospitalization (n/**%**)**					
Never	9	36,0%	9	36,0%	** 0.91**0^**α**^
5 years	3	12,0%	2	8,0%	
3 years	6	24,0%	5	20,0%	
This year	7	28,0%	9	36,0%	
**Vaso-occlusive crisis (n/**%**)**					
No	5	23%	7	28%	** 0,74**1^**β**^
Yes	20	77%	18	72%	
**Osteonecrosis (n/**%**)**					
No	23	92%	23	92%	** >0.99**9^**β**^
Yes	2	8%	2	8%	
**Leg lesion (n/**%**)**					
No	22	88%	24	96%	** 0.60**9^**β**^
Yes	3	12%	1	4%	

^*α*^Chi-square test. ^*β*^Fisher's test. ^*γ*^Mann-Whitney test. HU: hydroxyurea.

**Table 2:** Haematological parameters of patients with SCA during the trial.

**Haematological parameters**	**N **_**(placebo; study)**_		**Placebo Group**			**Study Group**			***p*-value**
			**M0**	**M2**	**M4**	**M0**	**M2**	**M4**	
**Erythrocytes x 10** ^**6**^ ** (/mm** ^**3**^ **)**	20	16	2.5	2.4	2.4	2.5	2.5	2.5	**0.378**
**Hb (g/dL)**	20	16	8.9	9.1	9.2	8.7	9.1	9.4	**0.525**
**HbF (g/dL)**	18	17	13.6	16.0	14.8	13.9	15.2	16.4	**0.455**
**HbS (g/dL)**	19	16	78.9	79.1	77.9	78.4	78.0	76.4	**0.882**
**Haematocrit (**%**)**	20	16	26.9	26.7	27.0	26.3	26.8	28.1	**0.304**
**MCV (fL)**	20	16	106.9	111.5	113.2	106.2	107.7	111.8	**0.480**
**MCHC (**%**) **	20	16	34.2	34.1	34.2	33.4	34.1	33.7	**0.661**
**Platelets x 10** ^**3 **^ **(/mm** ^**3**^ **)**	20	15	435	378	373	383	331	364	**0.551**
**Reticulocytes (**%**)**	18	18	11.6	11.8	8.9	9.1	9.1	7.4	**0.586**

Two-way repeated measures ANOVA: HbF (foetal haemoglobin), Hb (haemoglobin), HbS (haemoglobin S); MCV (mean corpuscular volume); MCHC (mean corpuscular haemoglobin concentration); SCA (sickle cell anaemia); M0 (timepoint before receiving placebo or L-arginine supplement); M2 (timepoint two months after receiving placebo or L-arginine supplement); M4 (timepoint four months after receiving placebo or L-arginine supplement).

**Table 3:** Frequency of pain in patients with SCA.

**Frequency of pain**	**Placebo group**			**Study group**			***p*-value**
	**M0 **	**M2 **	**M4 **	**M0 **	**M2 **	**M4 **	
**Never, n (**%**)**	7 (28)	7 (28)	7 (28)	6 (24)	6 (24)	6 (24)	**0,027** **∗**
**Every year, n (**%**)**	3 (12)	3 (12)	3 (12)	13 (52)	13 (52)	13 (52)	
**Every month, n (**%**)**	7 (28)	7 (28)	7 (28)	3 (12)	3 (12)	5 (20)	
**Every week, n (**%**)**	2 (8)	2 (8)	3 (12)	0 (0)	0 (0)	1 (4)	
**Every day, n (**%**)**	6 (24)	6 (24)	5 (20)	3 (12)	3 (12)	0 (0)	

Chi-square test: M0 (timepoint before receiving placebo or L-arginine supplement); M2 (timepoint two months after receiving placebo or L-arginine supplement); M4 (timepoint four months after receiving placebo or L-arginine supplement).

## Data Availability

The data are available on the website of the Brazilian Register of Clinical Trials at www.ensaiosclinicos.gov.br/rg/RBR-2t56nz/.

## References

[1] Sani M. A., Adewuyi J. O., Babatunde A. S. (2015). The iron status of sickle cell anaemia patients in ilorin, north central nigeria. *Advances in Hematology*.

[2] Morris C. R. (2014). Alterations of the arginine metabolome in sickle cell disease a growing rationale for arginine therapy. *Hematology/Oncology Clinics*.

[3] Belisário A. R., Silva C. M., Velloso-Rodrigues C. (2018). Genetic, laboratory and clinical risk factors in the development of overt ischemic stroke in children with sickle cell disease. *Hematology, Transfusion and Cell Therapy*.

[4] Yeung S. L. A., Lin S. L., Lam H. S. H. S. (2016). Effect of L-arginine, asymmetric dimethylarginine, and symmetric dimethylarginine on ischemic heart disease risk: A Mendelian randomization study. *American Heart Journal*.

[5] Elias D. B., Barbosa M. C., Rock L. B. (2013). L-arginine to an adjuvant drug in the treatment of sickle cell anemia. *British Journal of Haematology*.

[6] Tran H., Gupta M., Gupta K. (2017). Targeting novel mechanisms of pain in sickle cell disease. *Blood*.

[7] Morris C. R., Vichinsky E. P., Van Warmerdam J. (2003). Hydroxyurea and arginine therapy: Impact on nitric oxide production in sickle cell disease. *Journal of Pediatric Hematology/Oncology*.

[8] Sullivan K. J., Kissoon N., Sandler E. (2010). Effect of oral arginine supplementation on exhaled nitric oxide concentration in sickle cell anemia and acute chest syndrome. *Journal of Pediatric Hematology/Oncology*.

[9] Liu C., Wajih N., Liu X. (2015). Mechanisms of human erythrocytic bioactivation of nitrite. *The Journal of Biological Chemistry*.

[10] Lockamy V. L., Huang J., Shields H. (2003). Urease enhances the formation of iron nitrosyl hemoglobin in the presence of hydroxyurea. *Biochimica et Biophysica Acta (BBA) - General Subjects*.

[11] Khade R. L., Yang Y., Shi Y. (2016). HNO binding in heme proteins: effects of iron oxidation state, axial ligand, and protein environment. *Angewandte Chemie International*.

[12] Cokic V. P., Beleslin-Cokic B. B., Tomic M., Stojilkovic S. S., Noguchi C. T., Schechter A. N. (2006). Hydroxyurea induces the eNOS-cGMP pathway in endothelial cells. *Blood*.

[13] Charache S., Terrin M. L., Moore R. D. (1995). Investigators of the multicenter study of hydroxyurea in sickle cell anemia. Effect of hydroxyurea on the frequency of painful crises in sickle cell anemia. *New England Journal of Medicine*.

[14] Ballas S. K. (2012). More definitions in sickle cell disease: Steady state v base line data. *American Journal of Hematology*.

[15] Tsikas D. (2005). Methods of quantitative analysis of the nitric oxide metabolites nitrite and nitrate in human biological fluids. *Free Radical Research*.

[16] Elias D. B., Rocha L. B., Cavalcante M. B. (2012). Correlation of low levels of nitrite and high levels of fetal hemoglobin in patients with sickle cell disease at baseline. *Revista Brasileira de Hematologia e Hemoterapia*.

[17] Lopez B. L. (1996). Nitric oxide metabolite levels in acute vaso‐occlusive sickle‐cell crisis. *Academic Emergency Medicine*.

[18] Weiner D. L., Hibberd P. L., Betit P. (2003). Preliminary assessment of inhaled nitric oxide for acute vaso-occlusive crisis in pediatric patients with sickle cell disease. *Journal of the American Medical Association*.

[19] Morris C. R., Kuypers F. A., Lavrisha L. (2013). A randomized, placebo-controlled trial of arginine therapy for the treatment of children with sickle cell disease hospitalized with vaso-occlusive pain episodes. *Haematologica*.

[20] Cox S. E., Ellins E. A., Marealle A. I. (2018). Ready-to-use food supplement, with or without arginine and citrulline, with daily chloroquine in tanzanian children with sickle-cell disease: a double-blind, random order crossover trial. *The Lancet Haematology*.

[21] Bakshi N., Morris C. R. (2016). The role of the arginine metabolome in pain: Implications for sickle cell disease. *Journal of Pain Research*.

[22] Kato G. J., Hebbel R. P., Steinberg M. H. (2009). Vasculopathy in sickle cell disease: biology, pathophysiology, genetics, translational medicine, and new research directions. *American Journal of Hematology*.

[23] Manwani D., Frenette P. S. (2013). Vaso-occlusion in sickle cell disease: pathophysiology and novel targeted therapies. *Blood*.

[24] Kau D. K., Finnegan E., Barabino G. A. (2009). Sickle red cell-endothelium interactions. *Microcirculation*.

[25] Canalli A. A., Franco-Penteado C. F., Saad S. T. (2008). Increased adhesive properties of neutrophils in sickle cell disease may be reversed by pharmacological nitric oxide donation. *Haematologica*.

[26] Zhang D., Xu C., Manwani D. (2016). Neutrophils, platelets, and inflammatory pathways at the nexus of sickle cell disease pathophysiology. *Blood*.

[27] Villagra J., Shiva S., Hunter L. A. (2007). Platelet activation in patients with sickle disease, hemolysis-associated pulmonary hypertension, and nitric oxide scavenging by cell-free hemoglobin. *Blood*.

[28] Lundberg J. O., Weitzberg E., Gladwin M. T. (2008). The nitrate-nitrite-nitric oxide pathway in physiology and therapeutics. *Nature Reviews Drug Discovery*.

